# Does Believing in Fate Facilitate Active or Avoidant Coping? The Effects of Fate Control on Coping Strategies and Mental Well-Being

**DOI:** 10.3390/ijerph17176383

**Published:** 2020-09-02

**Authors:** Wesley C. H. Wu, Sylvia Xiaohua Chen, Jacky C. K. Ng

**Affiliations:** 1 Department of Applied Social Sciences, Hong Kong Polytechnic University, Hong Kong, China; wes_ch@hotmail.com; 2 Department of Counselling & Psychology, Hong Kong Shue Yan University, Hong Kong, China; ckng@hksyu.edu

**Keywords:** social axioms, fate control, locus of control, coping, mental well-being

## Abstract

The development of control-related constructs has involved different approaches over time, and yet internal and external locus of control are conceptualized as dichotomous factors influencing active versus avoidant coping strategies. While external control is associated with avoidance, a similar belief construct fate control, which denotes that life events are pre-determined and influenced by external forces but predictable and alterable, challenges the assumption of incompatibility between fate and agency. To develop a dynamic model of control, we suggest that external control would affect avoidant coping, which in turn would affect psychological distress, whereas fate control would affect both active and avoidant coping when dealing with stress. The model was supported among Hong Kong Chinese using a cross-sectional approach in Study 1 (*n* = 251) and hypothetical stressful scenarios in Study 2 (*n* = 294). The moderating effect of perceived controllability was observed in coping behaviors using a diary approach in Study 3 (*n* = 188). Our findings offer an alternative perspective to the dichotomous view of control and provide implications for coping strategies and mental well-being.

## 1. Introduction

Control is one of the central constructs in public health and has received extensive attention in the psychology literature. Theory and research have emphasized the importance of control in personality, social behavior, mental health, and physical well-being. Due to its broad conceptual bases, however, this generic construct has been studied under diverse theoretical frameworks. Skinner [1] identified more than 100 constructs that were related to control, such as cognitive control, decision control, learned helplessness, locus of control, relinquishment of control, retrospective control. The main objective of the present research is to examine a dynamic control construct, fate control, which offers an alternative perspective on the mental well-being outcomes of controllability.

Over the years, locus of control has been extended to the study of personality, clinical, developmental, and social psychology [2,3,4]. It refers to the extent to which individuals believe that the outcomes of events in their lives are influenced by internal or external forces [5]. Research has documented moderate heritability estimates of approximately 0.30 for locus of control [6], revealing both genetic and environmental influences on belief in control. In particular, additive genetic influences account for 31% of the variance in sense of personal control or lack of control over the direction of one’s own life and 34% in beliefs about how responsible people are for misfortunes in their lives [7]. Reviews and meta-analyses supported the positive impact of internal control, such as better academic achievement, favorable task performance and social experiences, greater job motivation, while external control results in negative consequences, including poor school achievement, learned helplessness, ineffective stress management and reduced self-evaluations [8,9,10].

A variety of studies show that locus of control is a strong predictor of mental well-being. Generally speaking, internal control is a protective factor in the prediction of mental health, while external control has negative impact on well-being. For example, when facing stress in life, depression in men was negatively correlated with internal locus of control [11]. In the workplace, external locus of control was negatively correlated with job satisfaction, physical well-being, and psychological well-being among employees in 24 countries around the world [12].

### 1.1. The Mediating Effects of Coping Strategies

While research has identified the relationship between locus of control and mental well-being [11,13], the underlying mechanism has not been fully examined in these studies. Other studies provide insight into understanding such mechanisms. Lefcourt [14] extended the concept of general expectancies of reinforcement [5] to the control over unpleasant stimuli. As Lefcourt [14] put it, “in being forced to hear predictable noise we may stop work and wait until it ceases, or steel ourselves for the onset, minimizing our own responses to the noise. We are not as helpless as we might otherwise be since we can do something to minimize the impact of the predictable noise. It is this perception of the ability ‘to do something’ that gives rise to the concept of perceived control” (p. 5). Reactions to unpleasant stimuli are not only shaped by individuals’ perceptions of the stimuli, but also the ability of individuals to cope with the stimuli. Following this view, Folkman and Lazarus [15] focused on individuals’ abilities to cope with the stimuli. They defined coping as “the cognitive and behavioral efforts made to master, tolerate, or reduce external and internal demands and conflicts among them” (p. 223).

There are different categorizations of coping strategies. One common categorization is problem-focused versus emotion-focused coping [15]. Coping strategies that aim to manage or alter the stressful environment are problem-focused coping, while those aiming to regulate the stressful emotions are emotion-focused coping. Individuals who appraise a situation as unalterable are more likely to use emotion-focused coping, whereas individuals who perceive an event as changeable are more likely to use problem-focused techniques [15]. This idea on appraisal and coping was supported in the three stages of a mid-term examination. Problem-focused coping was more prominent before the examination when students were able to adjust their study effort, while emotion-focused coping was more prominent when waiting for the results to be released.

Other researchers categorized coping using approach-avoidance distinction [16]. Active coping strategies are either behavioral or psychological responses designed to change the nature of the stressor itself or how one thinks about it, whereas people using avoidant coping strategies engage in activities (such as alcohol use) or mental states (such as withdrawal) that keep them from directly addressing stressful events [16]. Active coping strategies, whether behavioral or emotional, are associated with better adjustment when dealing with stressful events, because individuals exert some forms of personal control over the situation or their emotion. In contrast, avoidance may be functional in the short run, but maladaptive for long periods, exacerbating distress. Thus, uncontrollability induces negative emotions and results in psychological distress [17,18].

In Terreri and Glenwick’s [19] study among adolescents, positive religious coping (a type of active coping) was positively correlated with positive affect and life satisfaction, and was negatively correlated with depressive symptoms at times of stress. In addition, negative religious coping (a type of avoidant coping) was positively correlated with negative affect, depression and anxiety symptoms, and negatively correlated with positive affect and life satisfaction. In the medical domain, the relationship between appraisal and coping strategies in cancer patients was also identified [20]. Cancer patients who appraised their illness as a threat were more likely to use problem-focused coping (a form of active coping strategy). In contrast, individuals who appraised their cancer as a harm/loss were more likely to use avoidant coping strategies.

To further study coping strategies as mediators of the relation between personality traits and affect, Bartley and Roesch [21] found that active coping strategies mediated the relation between conscientiousness and positive affect. It was consistently shown that conscientiousness was correlated with internal locus of control [22,23], probably because both conscientiousness and internal control contain components of self-control and persistence. In this light, locus of control may work through coping strategies to affect psychological adjustment.

Empirically, the mediation effect of coping between control belief and adjustment to various stressful life events was supported in various domains [24,25,26,27]. For example, patients who received cancer treatment with higher levels of control over their emotional symptoms reported less avoidant coping, predicting lower levels of psychological distress [28]. Among sexual assault survivors, avoidant coping strategies (social withdrawal and problem avoidance) mediated the relations between control over the recovery process and distress. Specifically, survivors with higher levels of control over the recovery process experienced less distress because they tended to use less avoidant coping strategies [25].

In sum, locus of control is an important factor contributing to psychological well-being. In particular, the negative impact of external control is supported by previous research. Coping strategies can be one possible underlying mechanism explaining such relationship. Therefore, in this research we hypothesize coping strategies will mediate the effect of external control on mental well-being.

### 1.2. Believing in Fate and Fate Control

Fate denotes that the outcome of an event is predetermined [29]. Although Eastern culture (in particular Chinese) has a stronger belief in fate than Western culture [30], 75% of American undergraduates believe in fate [31]. In contrast to the conventional theories that regard fate as one of the external locus of control [5], research in recent decades has identified personal agency in belief in fate, namely fate control, which is the belief that life events are determined by impersonal, external forces; however, there is a possibility for individuals to alter the outcomes by their efforts [30]. The factor structure of fate control has been validated in multicultural studies conducted in over 40 nations [32]. Other researchers have proposed similar constructs, such as negotiable fate, representing the belief that individuals can negotiate with fate for control by exercising personal agency within the constraints bounded by fate [33], and malleable fate, which reflects an implicit theory about the nature of fate being changeable [34]. Empirically, believing that fate is controllable is also evident in Western culture, including the general public in the US [34] and European Americans [35].

The relationship between fate control and well-being measures is mixed. Some studies found that fate control was negatively related to well-being indicators and destructive behaviors, such as perceived stress [36], emotional rumination [37], general death anxiety [38], negative mood and problematic gambling [39]. However, fate control was also reported to be positively correlated with self-esteem, interpersonal harmony, and satisfaction about various life circumstances, including general life satisfaction, family situation, financial situation, interpersonal relationships, chosen profession/career, housing situation [40,41].

### 1.3. Fate Control and External Locus of Control

Fate is one of the determining forces in belief in external control, together with chance, luck and powerful others [42]. Thus, both external control and fate control share similarity in predicting coping responses. Believing in external control has detrimental effects because perceiving outcomes as uncontrolled by one’s responses will generate a sense of helplessness and hence the feeling of hopelessness [43,44,45]. Accordingly, coping efforts will focus on avoidance and detachment from stress. Avoidant coping tactics include venting of emotions (ventilating distress and negative feelings), behavioral disengagement (reducing efforts and even giving up goals), mental disengagement (distracting oneself from thinking about goals and efforts), and restraint coping (holding back and waiting without acting) [46].

At the same time, for people believing in fate control, attributing adversity to fate facilitates acceptance. Rothbaum, Weisz, and Snyder [47] pointed out that rather than relinquishing control, people sometimes adjust themselves to fit in the existing environment. By accepting that the outcome is beyond personal control, these individuals lower their expectation of success as well as the importance of meeting their goals [48], or by attributing failure to uncontrollable factors [49]. Bond and colleagues [50] found that people high in fate control were likely to use avoidant coping strategies, such as wishful thinking and distancing in the face of challenging life events. Therefore, we hypothesize that people high in fate control will tend to use avoidant coping, which is similar to external control.

In addition, fate control encompasses more than fatedness, as things can be done to modify one’s fate [30], such as feng shui. The distinction between fate control and external control was supported by empirical findings, albeit limited. A few studies reported the correlation between the two control beliefs in both Eastern and Western cultures [51,52]. Using internal–external scale [5], Singelis and colleagues [52] reported a correlation of 0.18 between the two beliefs in the US. Using the Chinese Personality Assessment Inventory (CPAI) [53], Chen and colleagues [51] reported a moderate correlation of 0.28 between fate control and external control in the internal versus external locus of control personality subscale in Chinese culture. The small but positive correlations in these two studies suggested the divergent validity between the two belief constructs.

### 1.4. Fate Control Can also Induce Adaptive Coping

Although both external control and fate control predict avoidant coping, their divergence may have implications for coping strategies. People high in external control believe that powerful others (including fate) determine their personal outcomes, and they are likely to relinquish personal effort. However, believing in fate control involves taking actions to improve fated outcomes. While external control is incompatible with personal choice, fate control can be a form of bounded agency, demonstrating personal effort in constrained situations [35]. Previous research has shown that believing in fate does not always reduce personal effort. In a large-scale community study in Romania, Dinca and Iliesca [40] found that people high in fate control reported more health-protective behaviors, including higher medication intake and more visitations to doctor than those low in fate control, despite the same reported health conditions. In another study on fate control and well-being among HIV patients, fate control was positively related to resilience, which in turn positively predicted well-being [54].

More studies have confirmed the positive linkage between fate control and personal effort in academic setting. For example, Zhou, Leung, and Bond [55] linked fate control to achievement-related indices at the culture level, and found it positively related to academic achievement and economic competitiveness. At the individual level, a positive relation between fate control and academic performance was identified among Hong Kong adolescents [56]. In addition, students believing in fate control also reported higher intention to study, so as to improve their academic outcomes [57]. These findings are aligned with a popular Chinese saying that “knowledge can change one’s fate”. In the same study, Liem and colleagues [57] also found that fate control was positively correlated with the intention to donate money to street children to improve the life of these children. While people high in fate control believed that adversity of street encountered by children was fated, they also believed that they could donate money to improve these children’s living conditions. In a community study in the US, Kim et al. [34] found that consumers believing that fate is malleable were more likely to choose an indulgent option as a coping strategy to compensate for an unfavorable day ahead. The above studies demonstrate that people believing in fate control also believe in personal agency when changes are possible.

In a study examining the relationship between social axioms [30], which refer to generalized beliefs about the world, and organizational commitment [58], young employees aged 18 to 25 were provided with a list of different human resource practices and were asked to indicate the extent to which each of these practices would make them feel committed to the organization. Five bundles of human resource practices were finally categorized by participants. Fate control predicted human resources practice bundle that focused on career development as well as incentives and recognition, indicating that people high in fate control believed that organizational efforts (e.g., providing more training, developmental opportunities, and other incentives) could contribute to greater commitment from their employees. These results show that people with high levels of fate control believe in not only personal agency, but also agency at the organizational level.

The above research highlights that people high in fate control also make efforts to improve their own lives or others’ lives. They do not merely accept one’s fate. In this sense, fate control can also be related to primary control (i.e., changing the environment so that it fits one’s need) [47]. Thus, fate control may have adaptive value for psychological functioning, which would explain the mixed findings on fate control and mental well-being. Therefore, we hypothesize that people high in fate control will be more likely to engage in active coping.

### 1.5. The Moderating Effect of Perceived Controllability

People high in fate control are hypothesized to engage in both active and avoidant coping. This conceptualization needs further examination to understand how the seemingly paradoxical forces of fate control work with or against each other to produce different outcomes in coping. As such, the next question to address is what factor makes fate control exhibit different coping responses to stressful situations.

We propose that the perceived control over stressful situations can moderate the effect of fate control on coping outcomes. Perception of control over a stressful situation arises from whether something can be done in the situation. Previous research shows that one’s appraisals of whether the stressful situation is controllable and whether there are sufficient resources to control the situation affect the choice of coping strategies [59]. When an individual appraises a stressful event as controllable, one tends to seek planning, strategize, take preventative efforts, and make direct action. In contrast, appraisal of low control leads to confusion, escape, pessimism, and passivity [60]. Recently, Frazier and colleagues [61] showed that the perceived control over the current stressful event was negatively correlated with avoidant coping but was positively correlated with positive reinterpretation, a form of active coping.

People high in fate control believe that life events are pre-determined but outcomes may be alterable. When a stressful situation is perceived to be high in controllability, people high in fate control may do something to exercise their control because of the belief that life events are malleable. In contrast, when a stressful situation is difficult to control (i.e., low in controllability), people believing in fate control may adapt to the fate belief and relinquish their personal effort, acting the same way as people who are high in external control. Therefore, avoidant type of coping will be adopted.

### 1.6. The Present Research

Based on the above conceptualizations, the present research aims to examine our hypothesized model explicating the pathways and consequences of external control and fate control: fate control positively predicts both active coping and avoidant coping; in contrast, external control positively predicts avoidant coping but negatively predicts active coping. Active coping and avoidant coping positively predict psychological adjustment and psychological distress, respectively. In terms of the strength of these associations, we expect to observe a small-to-medium effect size (i.e., *r* = 0.10 to 20) [62], which reflects a typical strength in social and personality psychology [63]. Furthermore, controllability of the stressors would moderate the effect of fate control on coping strategies. When controllability is high, fate control will predict more active coping and less avoidant coping. When controllability is low, fate control will predict less active coping and more avoidant coping.

In three studies recruiting different samples separately, multiple methods were used to examine the proposed framework. Study 1 adopted a cross-sectional design using a self-report approach to measure the impact of the belief systems on coping styles and general mental well-being. It was expected that external control would predict negative coping strategies and hence negative well-being outcomes. In contrast, fate control would predict both active and avoidant coping strategies. Study 2 employed hypothetical scenarios to assess coping patterns in specific situations. Participants were given the same set of hypothetical scenarios, so that the controllability of each scenario was equivalent across participants. Study 3 adopted a diary approach to gauge real life stressors and coping responses over time. It was hypothesized that controllability of stressors would moderate the effect of fate control, but not external control.

## 2. Study 1

To distinguish the two control beliefs (namely fate control and external control) and their divergent effects on coping styles and mental well-being, the hypothesized framework was tested with self-report measures using a cross-sectional design in the first study.

### 2.1. Method

#### 2.1.1. Participants and Procedure

Two hundred and fifty-one undergraduate students (173 females; *M_age_* = 21.05, SD = 1.69) responded to recruitment emails and completed an online survey in Chinese. In all studies reported in this paper, informed consent was obtained, and confidentiality was ensured at the beginning of each survey.

#### 2.1.2. Measures

Fate Control. The 8-item subscale of fate control was extracted from the Social Axioms Survey II (SAS-II) [64] (α = 0.81). The Social Axioms Survey is an instrument with pan-cultural factor structure that has been well validated in a large-scale study across 40 cultural groups, including Hong Kong [32]. A sample item is, “There are certain ways for people to improve their destiny.” Respondents rated each belief statement on a 5-point Likert scale ranging from 1 (strongly disbelieve) to 5 (strongly believe).

External control. The internal–external Scale [5] was used to measure the extent to which individuals believe that their decisions and life are controlled by environmental factors which they cannot influence (α = 0.74). The scale consists of 23 pairs of statements that respondents can choose. In each pair, one statement reflects the belief focusing on internal control (e.g., “People’s misfortunes result from the mistakes they make”), while another statement reflects the belief focusing on external control, such as chance, luck, other people’s control (e.g., “Many of the unhappy things in people’s lives are partly due to bad luck”). High scores indicted high external control.

Coping style. The 66-item Ways of Coping Questionnaire [65] was used to assess the thoughts and behaviors employed by respondents when facing stress. Participates indicated the extent to which they used each strategy in stressful situations and responded on 4-point Likert scales from 0 (not used) to 3 (used a great deal). Eight types of coping strategies have been identified, namely problem-focused coping, seeking social support, wishful thinking, detachment, focusing on the positive, self-blame, tension reduction, and keeping to self. Adopting the approach of a similar study by Au and colleagues [66], the 11-item problem-focused coping subscale (e.g., “Come up with a couple of different solutions to the problem”) and 7-item seeking social support subscale (e.g., “Talk to someone to find out more about the situation”) were considered as active coping strategy. On the other hand, the 5-item wishful thinking subscale (e.g., “I daydream or imagine a better time or place than the one I am in”) and the 6-item detachment subscale (e.g., “I feel that time will make a difference—the only thing to do is to wait”) were regarded as avoidant coping strategy. The alphas of problem-focused coping, seeking social support, wishful thinking and detachment subscales were 0.86, 0.78, 0.73 and 0.68, respectively.

Psychological adjustment. Two indicators were adopted to capture one’s psychological adjustment, namely subjective happiness and life satisfaction. Subjective happiness. The 4-item subjective happiness scale (SHS) [67] was used to assess global happiness and well-being of individuals (α = 0.85). A sample item is “Some people are generally very happy; They enjoy life regardless of what is going on, getting the most out of everything; To what extent does this characterization describe you?” Respondents rated each item on 7-point Likert scales ranging from 1 (very inaccurate) to 7 (very accurate). Life satisfaction. The 5-item satisfaction with life scale (SWLS) [68] and an additional item from the delighted–terrible scale (D–T Scale) [69] were used to measure an overall evaluation of one’s life. A sample item of the SWLS is “In most ways my life is close to my ideal”, and the question of the D–T scale is “How do you feel about your life as a whole?” Both were anchored on 7-point Likert scales with the SWLS ranging from 1 (strongly disagree) to 7 (strongly agree) and the D–T scale ranging from 1 (terrible) to 7 (delighted). The overall alpha of life satisfaction was 0.89.

Psychological distress. The 21-item depression anxiety stress scales (DASS) [70] was used to assess one’s negative emotional syndromes appeared over the past week. Respondents rated each item on 4-point Likert scales ranging from 0 (did not apply to me at all) to 3 (applied to me very much or most of the time). The DASS consists of three subscales, namely depression (e.g., “I felt that life was meaningless”), anxiety (e.g., “I was aware of the action of my heart in the absence of physical exertion, such as sense of heart rate increase, heart missing a beat”), and stress (e.g., “I was intolerant of anything that kept me from getting on with what I was doing”). The alphas of depression, anxiety, and stress subscales were 0.88, 0.84 and 0.84, respectively.

### 2.2. Results and Discussion

#### 2.2.1. Testing the Proposed Model

Descriptive statistics and bivariate correlations of the measures are summarized in Table 1. Given the well-acknowledged advantages over regression-based approach [71], structural equation modelling was adopted to test the proposed model with the corrections of measurement errors. In the measurement model, parceling technique [72] with three randomly grouped parcels per latent factor was employed on fate control and external control, while for active coping, avoidant coping, psychological adjustment, and psychological distress, each indicator was treated as a parcel. Assessment of a model having acceptable fit was based on multiple criteria [73], namely the comparative fit index (CFI) [74] >0.90, non-normed fit index (NNFI) [75] >0.90, root-mean-square errors of approximation (RMSEA) [76] <0.08.

**Figure 1:**
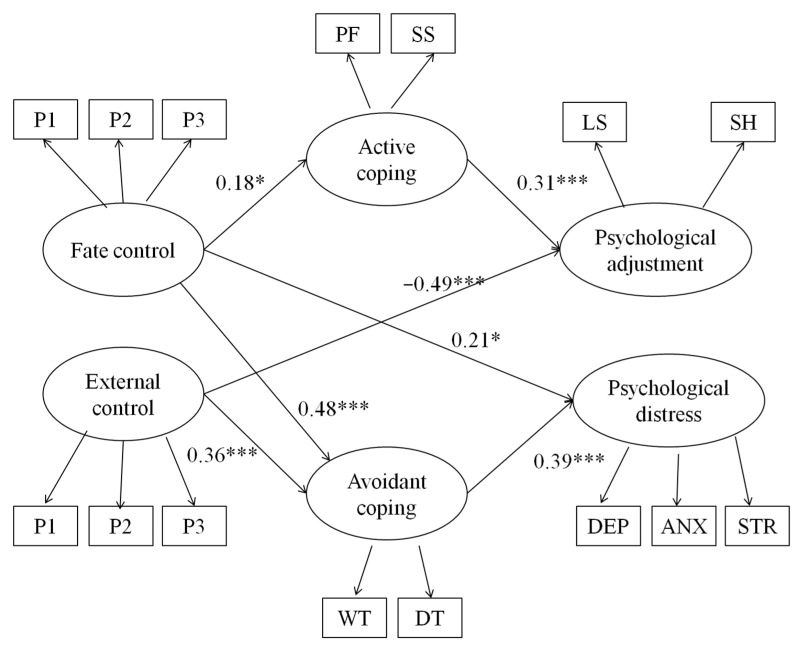
Structural equation model with standardized coefficients in Study 1. PF = problem-focused coping; SS = seeking social support; WT = wishful thinking; DT = distancing; SH = subjective happiness; LS = life satisfaction; DEP = depression; ANX = anxiety; STR = stress. All paths were tested. Only significant paths are shown. * *p* < 0.05, ** *p* < 0.01, *** *p* < 0.001.

Overall, the goodness of fit for the proposed model was adequate (Figure 1), with χ^2^ (76, *n* = 251) = 191.23, *p* < 0.001, CFI = 0.93, NNFI = 0.90, and RMSEA = 0.07. As hypothesized, fate control positively predicted both active coping (*b* = 0.11, β = 0.18, *p* = 0.03) and avoidant coping (*b* = 0.28, β = 0.48, *p* < 0.001). In addition, external control did not predict active coping significantly (*b* = −0.05, β = −0.02, *p* = 0.79), but positively predicted avoidant coping (*b* = 0.85, β = 0.36, *p* < 0.001). Active coping positively predicted psychological adjustment (*b* = 0.75, β = 0.31, *p* < 0.001), but not psychological distress (*b* = −0.09, β = −0.05, *p* = 0.48). In contrast, the effect of avoidant coping on psychological adjustment was not significant (*b* = −0.17, β = −0.07, *p* = 0.55), but it positively predicted psychological distress (*b* = 0.62, β = 0.39, *p* < 0.001).

#### 2.2.2. Testing the Mediating Effects of Coping Styles

From fate control to psychological adjustment and distress. On one hand, fate control positively predicted both active and avoidant coping styles, which in turn affected psychological adjustment and psychological distress, respectively. By a 95% bias-corrected bootstrap confidence interval based on 1000 bootstrap samples, the indirect effect was found to be significant from fate control to psychological distress through avoidant coping (b = 0.17, 95% bias-corrected bootstrap CI [0.05, 0.40]). The direct effect was not significant (b = 0.19, 95% bias-corrected bootstrap confidence interval (BCCI) [−0.001, 0.44]). This result indicated that avoidant coping fully mediated the effect of fate control on psychological distress. In contrast, the indirect effect from fate control to psychological adjustment through active coping (b = 0.08, 95% BCCI [−0.03, 0.32]) was not significant.

From external control to psychological adjustment and distress. External control positively predicted avoidant coping style, which in turn positively predicted psychological distress. The indirect effect was significant (b = 0.53, 95% BCCI [0.02, 3.54]), while the direct effect was not significant (b = 0.53, 95% BCCI [−0.12, 2.82]). This indicated that avoidant coping fully mediated the effect of external control on psychological distress.

Apart from examining the presence or absence of associations in the model, inspection of effect size r provided information on the strength of these associations. External control yielded an average effect size *r* of 0.20 and 0.18 on the two indicators of active coping and avoidant coping respectively, indicating an effect of medium size that might be of practical use in the short run [62]. Fate control yielded an effect of small size on the two indicators of active coping (an average effect size *r* of 0.11), and an effect that was large and might be consequential on the two indicators of avoidant coping in both the short and the long run (an average effect size *r* of 0.11). Both fate control and external control yielded effects that were large on most indicators of psychological adjustment and psychological distress (an average effect size *r* of 0.31), except that fate control yielded an effect of small size on the two indicators of psychological adjustment (an average effect size *r* of 0.10).

## 3. Study 2

Although the cross-sectional results in Study 1 have demonstrated the associations among the constructs in the proposed model, Study 1 did not specify stressful situations. People may adopt different coping strategies when dealing with different stressors [15]. Thus, Study 2 aimed to standardize stressful situations by presenting the same hypothetical stressful events to all participants, and asked them to report their coping strategies and their perceived effectiveness in reducing the stress [77].

### 3.1. Method

#### 3.1.1. Participants and Procedure

Two hundred and ninety-four undergraduate students (213 females, 1 unspecified; *M_age_* = 20.35, SD = 1.98) in Hong Kong responded to recruitment emails and completed an online survey in Chinese.

#### 3.1.2. Measures

Stressful scenarios and coping strategies. The hypothetical stressful scenarios were adopted from the extended Miller behavioral style scales [78]. The eight scenarios represented stress in different life domains, including work (layoff and business dinner), health (dentist, early cancer and terminal cancer), sports (ball game) and even life-and-death situations (hostage and plane). All participants were presented with eight stressful scenarios and instructed to vividly imagine encountering the situation. After reading each scenario, they were asked to write down what coping strategies they intended to employ to manage the stress associated with each scenario, and then indicated the extent to which the primary goal of their intended coping strategies was (a) to directly handle the demands/problems associated with the event in order to reduce its impact on them (i.e., problem-focused coping), (b) to reduce or manage their distress or uncomfortable feelings associated with the event (i.e., emotion-focused coping), and (c) to avoid the event (i.e., avoidant coping) on a 6-point Likert scale ranging from 1 (strongly disagree) to 6 (strongly agree). Active coping was computed by averaging the scores of problem-focused coping and emotion-focused coping, while avoidant coping was measured by the score of avoiding the event. Finally, participants were asked to rate the extent to which the adopted coping strategies could reduce their stress, on 7-point Likert scales ranging from −3 (largely increase the stress) to +3 (largely reduce the stress).

Fate control and external control. The same instruments used in Study 1 were adopted to measure fate control (α = 0.69) and external control (α = 0.73).

### 3.2. Results and Discussion

#### 3.2.1. Testing the Proposed Model

Overall scores of active coping, avoidant coping, and stress reduction were computed by averaging the corresponding scores across all eight scenarios, so as to reflect general coping responses across of different stressors.

**Figure 2:**
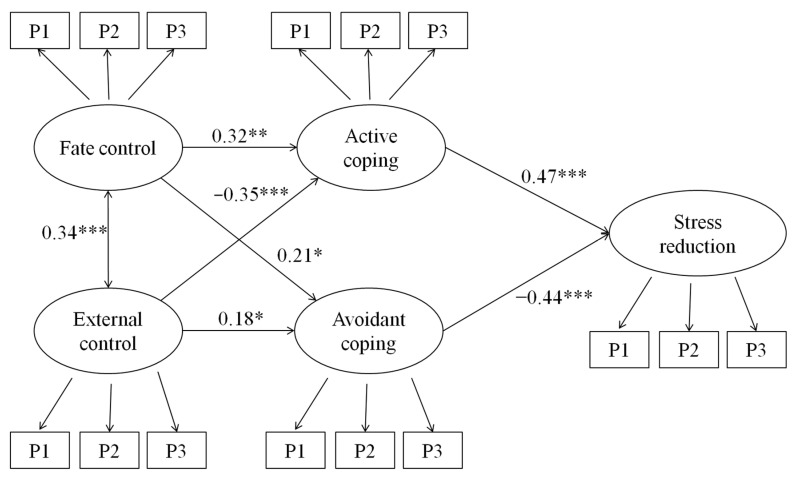
Structural equation model with standardized coefficients in Study 2. * *p* < 0.05, ** *p* < 0.01, *** *p* < 0.001.

Descriptive statistics and bivariate correlations of the measures are summarized in Table 2. Similar to Study 1, structural equation modeling was performed to test the proposed model with external control, fate control, active coping, avoiding coping, and stress reduction (Figure 2). Overall, the model fitted the data well, χ^2^ (81, *n* = 294) = 134.38, *p* < 0.001, CFI = 0.95, NNFI = 0.93, and RMSEA = 0.05.

Consistent with Study 1, fate control positively predicted both active coping (*b* = 0.34, β = 0.32, *p* < 0.001) and avoidant coping (*b* = 0.41, β = 0.21, *p* = 0.012), while external control negatively predicted active coping (*b* = −0.92, β = −0.35, *p* < 0.001) and positively predicted avoidant coping (*b* = 0.86, β = 0.18, *p* = 0.028). Finally, active coping positively predicted stress reduction (*b* = 0.88, β = 0.47, *p* < 0.001), while avoidant coping negatively predicted stress reduction (*b* = −0.45, β = −0.44, *p* < 0.001).

#### 3.2.2. Testing the Mediating Effects of Coping Styles

From fate control to stress reduction. Using a bootstrapping technique with 1000 bootstrap samples, indirect effect was found to be significant from fate control to stress reduction through active coping (*b* = 0.30, 95% BCCI [0.16, 0.57]) and from fate control to stress reduction through avoidant coping (*b* = −0.19, 95% BCCI [−0.43, −0.01]). The direct effect from fate control to stress reduction was found not significant (*b* = −0.10, 95% BCCI [−0.51, 0.24]). This result indicated that active and avoidant coping fully mediated the effect of fate control on stress reduction. Participants with high levels of fate control were likely to adopt both active and avoidant coping strategies, which in turn predicted stress reduction positively and negatively, respectively.

From external control to stress reduction. Similarly, indirect effect was found to be significant from external control to stress reduction through (negative) active coping (*b* = −0.81, 95% BCCI [−1.45, −0.43]), while the indirect effect from external control to stress reduction through avoidant coping was marginally significant (*b* = −0.39, 90% BCCI [−0.79, −0.05]). The direct effect of external control to stress reduction was not significant (*b* = −0.53, 95% BCCI [−1.31, 0.55]). This result indicated that (negative) active coping fully mediated the effect of external control on stress reduction. Participants with high levels of external control were less likely to adopt active coping, thereby less stress reduction.

Similar to Study 1, external control yielded the effect size *r* of 0.17 and 0.26 on active coping and avoidant coping respectively, indicating an effect of medium size [62]. Fate control yielded an effect of small size on active coping (0.10), and an effect of medium size on avoidant coping (0.19). Stress reduction was associated with fate control with a small effect-size *r* of 0.14, and external control with a large effect size *r* of 0.30.

Study 2 showed that the hypothesized effects of fate control were not only evident in general tendencies, but also in hypothetical stressors that are less common in everyday life (e.g., hostage, early caner and terminal cancer scenarios). Hence, Study 3 aimed to uncover whether people used different coping strategies in different situations through investigating the moderating effect of stress controllability.

## 4. Study 3

Study 1 was based on general behavioral tendencies, while Study 2 was based on a set of hypothetical scenarios. To test whether the model can be applied in actual situations, actual stressors and coping behaviors were examined in Study 3. A diary approach [21,79] was implemented to measure stressors and coping strategies adopted in daily life across multiple days. Thus, the role of coping as a mediator of the relations between control beliefs and well-being could be more rigorously evaluated.

### 4.1. Method

#### 4.1.1. Participants and Procedure

One hundred and eighty-seven university students (133 females; *M_age_* = 21.70, SD = 3.29) participated in Study 3. The student sample was recruited from local universities in Hong Kong. Participants responded to recruitment emails and took part in the survey in Chinese.

This study was divided into two phases: (a) the first day; and (b) the remaining twelve days. On the first day, participants were asked to complete a battery of questionnaire measuring their fate control, locus of control, and demographic information. About two weeks after the first session, participants were instructed to complete a short questionnaire for twelve days, with approximately two to three days per week for four consecutive weeks. On average, participants provided responses for 9.41 sessions out of the maximum of 12 sessions.

#### 4.1.2. Measures

Fate control and external control. The same instruments used in Study 1 were adopted to measure fate control (α = 0.78) and external control (α = 0.77).

Stressful events. In each session of the second phase, all participants were asked to recall and write down the most stressful event that happened to them in the past few days. They were then asked to rate the perceived controllability of the stressful event. Similar to Study 2, perceived controllability was rated on a 6-point Likert scale ranging from 1 (extremely uncontrollable) to 6 (extremely controllable).

Coping strategies. Similar to Study 2, after recalling and describing the stressful event, participants were asked to write down the coping strategies they had employed to manage and the primary goal of the coping strategies, except that they were only allowed to choose one of the three primary goals of their actions. When the intention of adopting an active or avoidant strategy (on a 6-point scale) was measured in Study 2, Study 3 tapped into actual coping strategies (single-answer question) due to different focuses in these two studies. In Study 2, stressors were based on hypothetic scenarios and participants were allowed to write down many coping strategies that they imagined they would employ, whereas Study 3 assessed daily life stressors participants experienced and their actual coping behavior.

### 4.2. *Results and Discussion*

The collected data had a multilevel structure with 1760 diary reports nested within 187 participants. To account for the nested structure, multilevel analyses were employed, controlling for age and gender. The analyses focused on the upper-level model, testing whether inter-individual differences in the overall perceived controllability across stressful events would moderate the effect of fate control on active (versus avoidant) coping strategies. Descriptive statistics and bivariate correlations of the measures on the upper-level model are summarized in Table 3. Specifically, fate control and external control yielded the small effect size *r* of 0.09 and 0.14, respectively, on active coping. Intraclass correlation of the outcome measure was 0.16, indicating that around 16% of the total variance in the intra-individual measure could be explained by the inter-individual differences. In the analysis of main effects, active coping was first regressed on fate control, external control and perceived controllability. First, perceived controllability significantly and positively predicted active (versus avoidant) coping (*b* = 0.09, *p* = 0.014, 95% CI [0.02, 0.16]), indicating that perceiving more control over stressful events predisposed individuals to cope with the events actively. Second, under the influences of perceived controllability, neither fate control nor external control could significantly predict active (versus avoidant) coping, *ps* > 0.05. To examine the moderating role of perceived controllability, we further regressed active (versus avoidant) coping on two two-way interaction terms, controlling for the main effects of fate control, external control, and perceived controllability. The results indicated that perceived controllability moderated the effect of fate control on active (versus avoidant) coping (*b* = 0.14, *p* = 0.003, 95% CI [0.05, 0.24]), while perceived controllability did not moderate the effect of external control on active (versus avoidant) coping (*b* = 0.001, *p* = 0.818, 95% CI [−0.01, 0.01]).

To interpret the moderation pattern of perceived controllability on active (versus avoidant) coping, the simple slope analysis was performed on the effects of fate control and active coping. The effect of fate control was delegated at two values of perceived controllability (1 SD above mean value and 1 SD below mean value). For those who perceived high controllability across stressful events (1 SD above mean value), fate control positively and marginally predicted active (versus avoidant) coping (*b* = 0.07, *p* = 0.055, 90% CI [0.01, 0.12]). Among those who perceived low controllability across stressful events (1 SD below mean value), fate control significantly and negatively predicted active (versus avoidant) coping (*b* = −0.09, *p* = 0.005, 95% CI [−0.15, −0.03]).

Taken together, the results of Study 3 supported our hypothesis that controllability of the stressors moderated the effect of fate control on coping strategy. Specifically, when the stressors were more controllable, people believing in fate control were more likely to use active coping strategies. This interaction effect was significant only for fate control but not for external control.

## 5. General Discussion

The present research aimed to understand the relationship between control and response to stress in the prediction of mental well-being. We proposed a model to differentiate two control belief systems, i.e., external control and fate control, and predicted their behavioral and psychological consequences in stressful situations. Results showed that while external control was positively related to avoidant coping, fate control was positively related to both active and avoidant coping. In addition, controllability of the stressors moderated such relationship. Specifically, facing stressors that were high in controllability, people high in fate control were more likely to adopt active coping. However, when the stressors were less controllable, people were less likely to adopt adaptive coping. This interaction was only significant for fate control but not external control.

### 5.1. Differentiating External Control and Fate Control

As beliefs about the nature of the world, both external control and fate control denote that life events are determined and controlled by forces outside the self. Aligned with previous studies, a slight positive correlation between external control and fate control was observed across our three studies (*r* = 0.25, 0.24, and 0.25, *ps* < 0.001, in Studies 1, 2, and 3, respectively). The findings from the cross-sectional design in Study 1 and the hypothetical scenarios design in Study 2 basically support the linking of the two control beliefs and coping strategies. Individuals endorsing fate control belief and external control belief are more likely to use avoidant coping. However, positive linkage between fate control and active coping is also identified, differentiating fate control from external control.

The concept that a belief composing of both life events are predetermined (determinism) and yet there are ways that individuals can exert influence over or shape their outcomes (alterability) seems counter-intuitive. However, such “conflation of belief types” co-exists. Burrus and Roese [31] showed that 75% people believe in fate, and 85% further believe that it is possible for events to be determined jointly by fate and individual action. Previous research found that fate control leads to avoidance [50] and that fate control induces personal agency [55,57]. These studies demonstrated either the determinism aspect or the alterability aspect of fate control. The present research has reconciled these opposing effects in a single model and found that people high in fate control tend to use both active and avoidant coping.

People believing in fate control score higher in both active and avoidant coping, indicating that they tend to use various coping strategies in their daily life. Ability to adapt different coping strategies across situations is one kind of coping flexibility [80]. Coping flexibility reflects an individual’s attitude toward how to cope effectively in situations and his/her intentions to display behaviors that are appropriate to the situation [78]. The present research found that people high in fate control tend to use different strategies, depending on how they perceive the stressors. Specifically, when the stressors are more controllable, they prefer active coping; in contrast, when the stressors are less controllable, they use less active coping. Compared with fate control believers, people high in external control have stronger preference for avoidant coping but not active coping, regardless of the controllability of the stressors.

### 5.2. Enriching the Understanding of Control Belief and Personal Agency

The relationship between control belief and personal agency has been well studied in the literature. People who believe in external control do not think that their actions bring desired outcomes, and therefore they are more likely to relinquish control and avoid stressful events [81]. The linkage between external control and avoidance has been established in various domains. In research on workplace stress, an internal control predicted help-seeking and positive thinking, while external control predicted the use of avoidant coping. In particular, powerful others predicted avoidance/resignation and chance predicted alcohol use [82]. Similar results were found among mid-level managers as well. Managers who reported higher external locus of control were more likely to have the wish to quit their jobs while those reported higher internal control were more likely to develop citizenship behaviors [83].

The present research provides an alternative perspective to this linkage between externality belief and personal agency. Although people endorsing fate control belief think that life is pre-determined, they believe that there are ways for people to influence the negative impact of fate. A high level of fate control is positively correlated with having a lucky number and reading one’s horoscope [52], both aiming to increase a sense of control over one’s future. Some researchers argued that believing in a lucky number or preferring a lucky number is an illusion of control [84]. In lottery, people prefer to choose their own random number instead of having others choose for them. Langer [84] allowed half of the participants to choose their own lottery ticket while the other half was randomly given a lottery ticket. When asked about repurchasing the ticket before the lottery outcome was announced, those who were allowed to choose their own numbers demanded more money than those who were given a random lottery ticket. Other researchers suggested that the illusion of control increased when the game was related to skills [85,86]. In sum, people high in fate control are likely to have a lucky number [52], which increases the sense of control.

The above research shows that people take actions to promote fortune or to avoid bad luck whenever the situation is controllable and action is possible. Given that people high in fate control are more likely to engage in these behaviors, they are likely to exhibit personal agency when the situation is controllable. Hence, fate control is more than merely “illusion of control”. It also induces actions to foster personal control. For example, people high in fate control have higher intention to study to improve their academic performance and to donate money to street children to improve the lives of these children [57].

### 5.3. Advancing the Understanding of Control and Mental Well-Being

The present research demonstrates that external control is negatively correlated with psychological adjustment (e.g., subjective happiness and life satisfaction) and is positively correlated with psychological distress (e.g., depression, anxiety, and perceived stress). In addition, such a relationship between external control and psychological adjustment is mediated by active coping and avoidant coping.

The negative linkage between external control and psychological functioning has been discussed in psychological theories and supported by empirical studies. For example, Seligman’s [45] learned helplessness theory explains the detrimental effects of external control because it reduces people’s propensity to engage in problem-solving activities and elicits depressive symptoms. Depressed people tend to perceive life events as uncontrollable and future action as futile [87]. As Maier and Seligman [88] pointed out, helplessness does not arise from the traumatic events per se, but from the belief about whether the organism can do something about them. It is not adversity itself but learning its uncontrollability that causes a sense of helplessness. Meta-analyses have revealed moderately strong correlations between external locus of control and depression [89]. In addition to depression symptoms, a recent meta-analysis by Cheng, Cheung, Chio, and Chan [90] also identified the moderately positive linkage between external locus of control and anxiety symptoms based on 152 studies in non-clinical populations.

Compared with external control, the relationship between fate control and psychological adjustment is not as straightforward. Firstly, fate control predicted both active coping and avoidant coping. However, since active coping positively predicted psychological adjustment while avoidant coping negatively predicted psychological adjustment, the overall effect of fate control to psychological adjustment became neutral. In Study 1, the total indirect effect from fate control to psychological adjustment was not significant (*b* = −0.04, *β* = −0.04, *p* = 0.59). Similarly, in Study 2, the total indirect effect from fate to stress reduction was not significant (*b* = 0.12, *β* = 0.06, *p* = 0.28). Fate control can foster the sense of personal action but also increase the burdens if the outcomes of some life events are not consistent with one’s wish. In this light, fate control can be both beneficial and detrimental to mental well-being, yielding to a non-significant relationship.

Future research is needed to investigate the origin of fate control belief. Chaturvedi and colleagues [33] suggested that people suffer from immutable constraints (e.g., countries with lower GDP and people in lower literacy levels) develop higher levels of negotiable fate, implying that socio-cultural context affects the prevalence of belief in fate. However, the present research found that there is variability in fate control across university students in Hong Kong. Hence, future research can be conducted to explore which individual factors, apart from socio-cultural factors, affect the development of fate control. Chen and colleagues [91] found that endorsement of reward for application and social cynicism is associated with experiences of family dysfunction. Other psychological and demographic factors can be explored to understand the origin of belief in fate control. In the three different samples of the present research, there are more female than male participants. It is unclear to what extent the current results are affected by the gender ratio of the samples, which needs further investigation.

Future studies may also examine the current model in different cultures. Cheng and colleagues’ [90] meta-analysis found the moderating effect of culture on the relationships between locus of control and psychological symptoms, such that the negative effects of external control were weaker in collectivistic cultures. The notion of external control is less negative in collectivistic cultures, probably because people can still have some personal control despite believing in fate. Testing the model in individualistic cultures will facilitate the understanding of how fate control influences the action-outcome contingency.

## 6. Conclusions

Since the development of social axioms [30], fate control has received relatively less attention than other axioms, such as social cynicism and reward for application [92], because of its ambiguity [93]. However, fate control is still an important factor to understand how people conceive of the world [64,94]. Fate control is not merely control by fate as an external force, but control through human strivings to maximize their outcomes in a contingent world [95]. By developing a dynamic model of control, this research has shown that external control predicts avoidant coping, which in turn predicts psychological distress, whereas fate control affects both active and avoidant coping when dealing with stress. Perceived controllability moderates their relationship, such that people believing in fate control are more likely to adopt active coping when stressors are controllable, but less likely to adopt active coping when stressors are low in controllability. The effect sizes of these associations and paths vary across dependent variables and assessment methods. The findings further our understanding of control beliefs, coping strategies, and mental health, but they are based on self-reports in Hong Kong context. Future research should examine this model in other cultures and adopt gender-balanced samples using experimental and longitudinal designs.

## Figures and Tables

**Table 1:** Descriptive Statistics and Bivariate Correlations among the Measures in Study 1.

	Mean (SD)	1	2	3	4	5	6	7	8	9	10
1. FATC	3.13 (0.61)	-									
2. ELOC	13.50 (4.15)	0.25 ***	-								
3. PFC	1.64 (0.45)	0.13 *	−0.19 **	-							
4. SS	1.49 (0.51)	0.08	−0.21 **	0.51 ***	-						
5. WT	1.57 (0.56)	0.24 ***	0.17 **	0.18 **	0.22 ***	-					
6. DT	1.39 (0.48)	0.40 ***	0.18 ^†^	0.28 ***	0.27 ***	0.51 ***	-				
7. SH	4.46 (1.13)	−0.13 *	−0.35 ***	0.16 *	0.18 **	−0.22 ***	−0.03	-			
8. LS	4.45 (1.07)	−0.07	−0.33 ***	0.29 ***	0.31 ***	−0.10	0.10	0.64 ***	-		
9. DEP	0.75 (0.64)	0.27 ***	0.31 ***	0.05	−0.01	0.30 ***	0.34 ***	−0.46 ***	−0.34 ***	-	
10. ANX	0.61 (0.56)	0.30 ***	0.30 ***	0.04	0.08	0.34 ***	0.36 ***	−0.40 ***	−0.24 ***	0.80 ***	-
11. STR	0.95 (0.62)	0.28 ***	0.34 ***	0.12	0.110	0.35 ***	0.35 ***	−0.41 ***	−0.25 ***	0.76 ***	0.81 ***

FATC = fate control; ELOC = external control; PFC = problem-focused coping; SS = seeking social support; WT = wishful thinking; DT = distancing; SH = subjective happiness; LS = life satisfaction; DEP = depression; ANX = anxiety; STR = stress. ^†^ *p* < 0.10, * *p* < 0.05, ** *p* < 0.01, *** *p* < 0.001.

**Table 2:** Descriptive Statistics and Bivariate Correlations among the Measures in Study 2.

	Mean (SD)	1	2	3	4	5	6
1. FATC	3.10 (0.52)	-					
2. ELOC	12.85 (4.07)	0.24 ***	-				
3. STR	4.64 (0.56)	0.11 ^†^	0.15 *	-			
4. CTRL	3.24 (0.50)	−0.10	−0.35 ***	−0.27 ***	-		
5. ACT	4.33 (0.52)	0.10 ^†^	−0.17 **	0.28 ***	0.18 **	-	
6. AV	3.12 (0.85)	0.19 **	0.26 ***	0.34 ***	−0.25 ***	0.05	-
7. SR	4.64 (0.56)	−0.14 *	−0.30 ***	−0.07	0.38 ***	0.34 ***	−0.34 ***

FATC = fate control; ELOC = external control; STR = perceived stress; CTRL = perceived controllability; ACT = active coping; AV = avoidant coping; SR = stress reduction. The scores of ACT, AV and SR were averaged across the eight scenarios. ^†^ *p* < 0.10, * *p* < 0.05, ** *p* < 0.01, *** *p* < 0.001.

**Table 3:** Descriptive Statistics and Bivariate Correlations among the Measures in Study 3.

	Mean (SD)	1	2	3
1. FATC	3.04 (0.56)	-		
2. ELOC	13.06 (4.32)	0.20 **	-	
3. CTRL	3.82 (0.53)	−0.01	−0.24 **	-
4. ACT	0.90 (0.12)	−0.09	−0.14 ^†^	0.40 *

FATC = fate control; ELOC = external control; CTRL = perceived controllability; ACT = active coping. ^†^ *p* < 0.10, * *p* < 0.05, ** *p* < 0.01.
